# Erroneous energy-generating cycles in published genome scale metabolic networks: Identification and removal

**DOI:** 10.1371/journal.pcbi.1005494

**Published:** 2017-04-18

**Authors:** Claus Jonathan Fritzemeier, Daniel Hartleb, Balázs Szappanos, Balázs Papp, Martin J. Lercher

**Affiliations:** 1 Institute for Computer Science and Cluster of Excellence on Plant Sciences, Heinrich Heine University, Düsseldorf, Germany; 2 Synthetic and Systems Biology Unit, Institute of Biochemistry, Biological Research Centre of the Hungarian Academy of Sciences, Szeged, Hungary; The Pennsylvania State University, UNITED STATES

## Abstract

Energy metabolism is central to cellular biology. Thus, genome-scale models of heterotrophic unicellular species must account appropriately for the utilization of external nutrients to synthesize energy metabolites such as ATP. However, metabolic models designed for flux-balance analysis (FBA) may contain thermodynamically impossible energy-generating cycles: without nutrient consumption, these models are still capable of charging energy metabolites (such as ADP→ATP or NADP^+^→NADPH). Here, we show that energy-generating cycles occur in over 85% of metabolic models without extensive manual curation, such as those contained in the ModelSEED and MetaNetX databases; in contrast, such cycles are rare in the manually curated models of the BiGG database. Energy generating cycles may represent model errors, *e*.*g*., erroneous assumptions on reaction reversibilities. Alternatively, part of the cycle may be thermodynamically feasible in one environment, while the remainder is thermodynamically feasible in another environment; as standard FBA does not account for thermodynamics, combining these into an FBA model allows erroneous energy generation. The presence of energy-generating cycles typically inflates maximal biomass production rates by 25%, and may lead to biases in evolutionary simulations. We present efficient computational methods (i) to identify energy generating cycles, using FBA, and (ii) to identify minimal sets of model changes that eliminate them, using a variant of the GlobalFit algorithm.

## Author summary

Genome-scale metabolic models are routinely used to simulate the growth of unicellular organisms, and are likely to become an important tool in the medical sciences. The most popular method employed for this task is flux balance analysis (FBA), a simplified mathematical description able to describe the simultaneous activity of hundreds of biochemical reactions. Cellular functions are often dependent on the availability of sufficient energy, and thus a correct representation of energy metabolism appears crucial to metabolic modeling. However, we found that the majority of FBA models generated directly from genome sequences, as well as a minority of carefully curated models, are capable of generating energy out of thin air. These models charge energy metabolites such as ATP without any nutrient uptake. We named the corresponding sets of reactions “erroneous energy generating cycles” (EGCs) and developed a high-throughput algorithm for their identification. We found EGCs in 238 (68%) of 350 metabolic models from three different databases. We developed a second, fully automated method for EGC removal. Simulations on the corrected models typically showed growth rates that were 25% slower than in the original models, demonstrating the importance of checking metabolic model reconstructions for EGCs.

## Introduction

Constraint-based analysis, in particular flux-balance analysis (FBA), is the current state of the art in genome-scale metabolic modeling [1]. Constraint-based modeling assumes a steady state (*i*.*e*., every internal metabolite that is produced must be consumed at the same rate) and imposes lower and upper bounds on metabolic fluxes. However, constraint-based analyses typically do not explicitly consider thermodynamics. As a result, the mathematical solution of constraint-based problems is often thermodynamically infeasible [2, 3]. Specifically, internal cycles (sometimes called type-III pathways [4]), which consist only of internal reactions and do not exchange metabolites with the environment, violate the second law of thermodynamics. The thermodynamic driving forces around a biochemical reaction cycle must add up to zero; hence, there cannot be a flux in a closed cycle [5–7].

**Fig 1:**
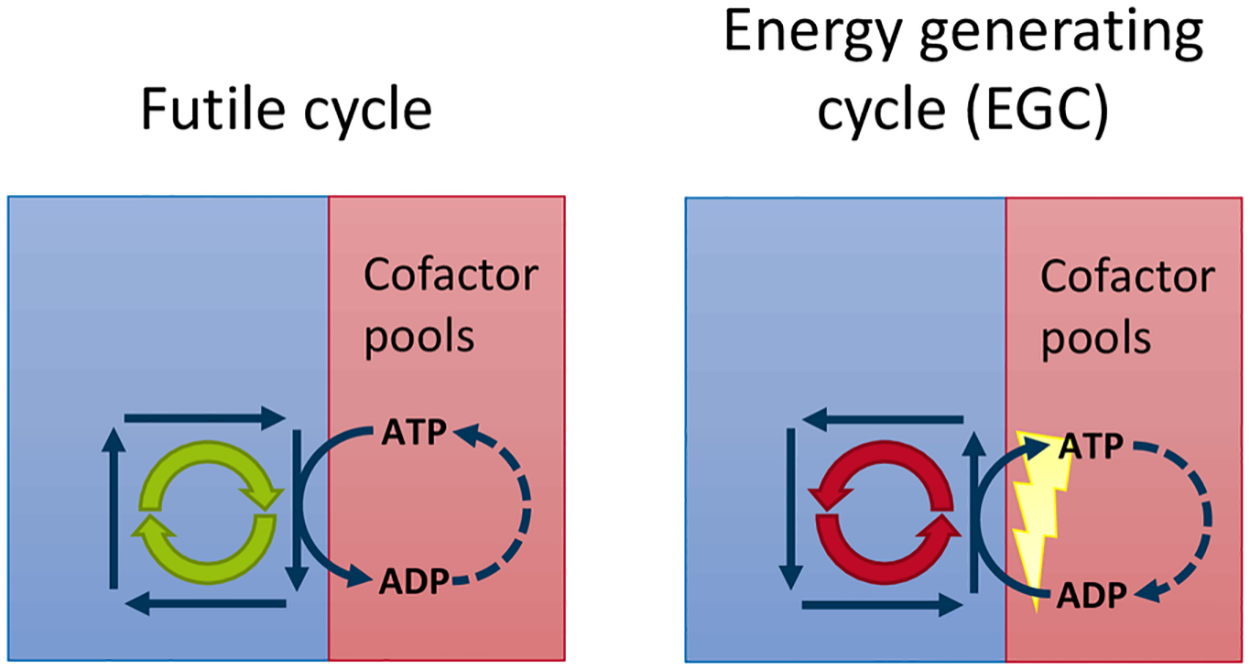
A futile cycle that consumes energy drawn from a cofactor pool (left) and an energy generating cycle (EGC) (right), which is thermodynamically impossible but occurs in some metabolic network models (figure extended from [12]). We can convert the type-II pathways to type-III pathways by closing the cycles in the cofactor pools (dashed arrows).

These thermodynamically infeasible type-III pathways [4] have to be distinguished from futile cycles (type-II pathways; Fig 1), which additionally consume cofactors to generate a driving force around the cycle [8, 9]. Futile cycles are not an artifact of metabolic modeling, but have been experimentally observed [10]; *e*.*g*., some prokaryotes that live in very energy-rich environments need to dissipate energy by converting ATP to ADP [11].

A futile cycle running in reverse would charge energy metabolites such as ATP without an external source of energy (Fig 1). Accordingly, we classify type-II pathways [12] into two subgroups by taking the directionality of cofactor utilization into account: (a) futile cycles, which consume energy and are thus thermodynamically feasible, and (b) energy generating cycles (EGC), which charge energy metabolites without a source of energy.

**Fig 2:**
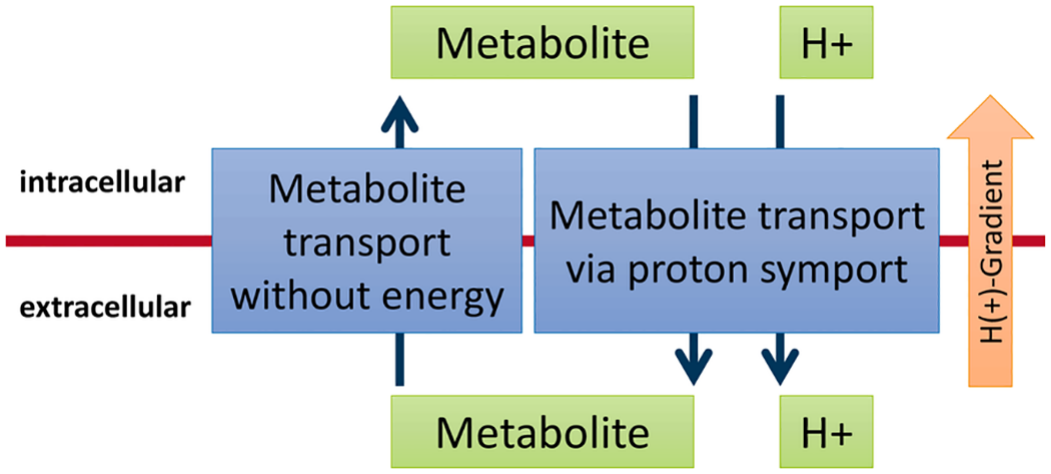
A simple (hypothetical) example of an energy generating cycle (EGC). A symporter that exports a metabolite and a proton acts together with a transporter that takes the same metabolite up without a proton. A combination of both reactions builds up a proton gradient that can then be utilized to generate energy (*e*.*g*., via an ATP synthase).

While such EGCs are thermodynamically impossible, they can—and, as we show below, do—occur in constraint-based models. Futile cycles will rarely occur in FBA solutions, as they dissipate energy and hence divert metabolic investment away from biomass production. EGCs, in contrast, can have a substantial effect on the predictions of constraint-based analyses, as they generate energy out of nothing that then supports *in silico* growth. A simple example illustrating a (hypothetical) EGC is shown in Fig 2.

Eliminating EGCs is crucial for the correct modeling of energy metabolism, as has been recognized earlier (see, *e*.*g*., [13–15]). While thermodynamically infeasible type-III pathways (internal cycles) can be easily removed through a simple post-processing step [5, 16], the same strategy cannot be used to suppress EGCs. In principle, EGCs could be excluded from the solution space by systematically assigning sufficiently detailed thermodynamic constraints. Thermodynamics-Based Metabolic Flux Analysis (TMFA) [17], for example, searches for a set of feasible metabolite concentrations such that all reactions proceed in the direction of negative free energy change (ΔG<0) or, equivalently, a ratio of product to substrate concentrations below the reaction’s equilibrium constant, *K*_*eq*_. However, it can be shown mathematically that for any flux distribution without type-III pathways, there exists a distribution of metabolite concentrations such that the flux distribution is thermodynamically feasible, *i*.*e*., all fluxes proceed in the direction of negative free energy change (see the theorem in [16]). This theoretical result respects the fact that metabolite concentrations must have a single value for all reactions they participate in; Supplementary S1 Text shows a small example network that illustrates the inability of ll-COBRA and TMFA to reliably exclude EGCs.

The simplest EGC could be established through an ATP energy dissipation reaction (ATP + H_2_O → ADP + Pi + H^+^) that is allowed to proceed in the backwards direction. An energy-generating backward flux can be achieved as long as the concentration ratio ([ATP][H_2_O]) / ([ADP][Pi][H^+^]) is smaller than the corresponding equilibrium constant *K*_*eq*_ = 2×10^-5^M^-1^. If we treat the concentration of H_2_O (55M) as constant, this cannot occur within the physiological concentration bounds assumed by Henry *et al*. [17], 10^-5^M and 0.02M, showing that TMFA’s metabolite concentration bounds avoid the utilization of at least some EGCs. Note, however, that reactions central to an EGC may have equilibrium constants compatible with the concentration bounds, especially if the total free energy change is spread over several individual reactions. Moreover, the TMFA strategy relies on the availability of equilibrium constants for all reactions central to the EGC.

In contrast to TMFA, several alternative thermodynamically informed constraint-based methods only consider chemical potentials, which do not incorporate information on reaction specifics such as the equilibrium constant *K*_*eq*_ [7, 12, 18, 19]. For every flux distribution free of type-III pathways, it is possible to find a distribution of chemical potentials such that all fluxes proceed in the direction of chemical potential reduction [16]. This means that potentials capable of driving energy dissipation reactions towards the high-energy metabolite can always be found. Thus, constrained-based methods designed to ensure thermodynamic feasibility based on freely variable chemical potentials do not guarantee the elimination of EGCs.

The detection and removal of EGCs is currently not part of established metabolic network reconstruction pipelines [2]. In particular, automatic reconstructions algorithms [20, 21] currently do not test for EGCs. Sometimes, EGCs are identified in the manual reconstruction process, and parts of the cycles are constrained to zero flux as a makeshift correction [13]. Accordingly, as we demonstrate below, the problem of erroneous free energy generation occurs in a majority of automated and a subset of manual network reconstructions.

## Results and discussions

### Erroneous energy-producing cycles occur in many published reconstructions

EGCs can be identified through a variant of FBA [14]. To efficiently identify the existence of diverse EGCs, we first add a dissipation reaction to the metabolic network for each metabolite used to transmit cellular energy; *e*.*g*., for ATP, the irreversible reaction ATP + H_2_O → ADP + P + H^+^ is added. These dissipation reactions close any existing energy-generating cycles, thereby converting them to type-III pathways. Fluxes through any of the dissipation reactions at steady state indicate the generation of energy through the metabolic network. Second, all uptake reactions are constrained to zero. The sum of the fluxes through the energy dissipation reactions is now maximized using FBA. For a model without EGCs, these reactions cannot carry any flux without the uptake of nutrients.

We used this approach to identify the presence of EGCs for 14 different energy metabolites (ATP, CTP, GTP, UTP, ITP, NADH, NADPH, Flavin adenine dinucleotide, Flavin mononucleotide, Ubiquinol-8, Ubiquinol-8, 2-Demethylmenaquinol 8, Acetyl-CoA, L-Glutamate) and for proton exchange between periplasm and cytosol (for simplicity counted as a 15^th^ “energy metabolite” below); see Suppl. S1 Table for the corresponding dissipation reactions. We did not require the energy dissipation reactions to be charge-balanced; e.g., in the reaction NADH → NAD^+^ + H^+^, we omitted the molecule that acts as the acceptor of the two electrons. Adding the electron acceptor to the dissipation reaction would not dissipate the energy stored in NADH, as this energy could then potentially be re-used by internal cofactor regeneration reactions; in this case, the dissipation reaction could be active even in the absence of EGCs. FBA models do not keep track of metabolite charges, and thus the general problem posed by charge unbalanced reactions is not that they affect constraint-based simulations directly; instead, they are a sign of incorrect reaction stoichiometry, which is especially severe in the case of electron imbalances. It is important that models are mass and electron balanced [2] before conducting the EGC analysis. While EGCs induced by mass or electron unbalanced reactions may be detected by our method, they cannot be removed properly without fixing the reaction stoichiometries.

**Fig 3:**
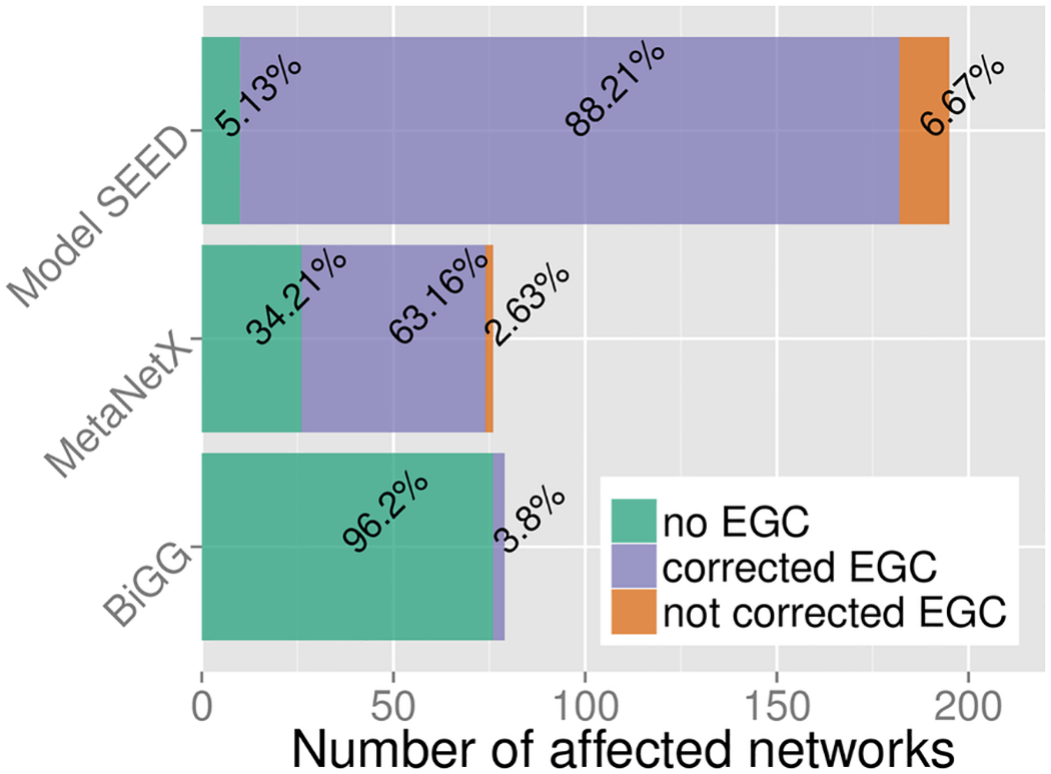
The majority of metabolic network reconstructions in two of the examined databases (ModelSEED and MetaNetX) contain erroneous internal EGCs that generate energy. In contrast, most models in BiGG do not contain EGCs. Total bar size reflects the number of models contained in each database. Green: models without EGCs; purple: models with EGCs that could be corrected through GlobalFit; orange: models with EGCs that cannot be corrected through reaction removals.

We analyzed all models in three large databases of constraint-based metabolic networks: BiGG [22], ModelSeed [23], and MetanetX [24]. Overall, we found that over two thirds (68%) of tested models supported a non-zero flux through at least one of the 15 energy dissipation reactions, although this percentage differed drastically between databases (Fig 3).

The BiGG database contains high-quality manual [2] and, in the case of 54 *E*. *coli* strains, semi-automated [25] genome scale metabolic reconstructions. We found EGCs in only 3 out of the 79 BiGG models (3.8%; Suppl. S2 Table). The ModelSEED database is connected to a service for high-throughput reconstruction and analysis of metabolic networks. A special feature is the fully automated reconstruction of genome-scale networks from genome sequences. Due to the fully automated reconstruction, models created by this service should be considered as draft models, and manual steps for model improvement are recommended [23]. Consistent with this recommendation, we identified EGCs in 95% of ModelSEED models (185 out of 195; Suppl. S2 Table). Finally, MetaNetX is a Meta-Database for metabolic network models, gathering metabolic networks from different databases (including The ModelSEED and the BiGG database) and mapping them to one common namespace. This allows easy meta-analysis, manipulation, and comparison of those models [24]. Our FBA strategy found EGCs in 66% of MetaNetX models (50 out of 76; Suppl. S2 Table).

### GlobalFit can eliminate >90% of EGCs by removing reactions

For each network with EGCs, we then used a slightly modified version of GlobalFit [26] to suggest a minimal number of reaction removals that eliminate all EGCs, allowing independent removals of forward and backward directions for reversible reactions. GlobalFit was originally designed to reconcile inconsistencies between FBA model predictions and measured growth/non-growth data, *e*.*g*., from gene knockouts. GlobalFit uses a bi-level optimization method to identify the minimal set of network changes needed to correctly predict all experimentally observed growth and non-growth cases (or a subset thereof) simultaneously. We slightly altered the original algorithm, now simultaneously contrasting one growth case (the network with the biomass reaction as the objective function, ensuring that the suggested modifications do not interfere with biomass production), and one non-growth case (the network with the sum of energy dissipation reactions as the objective function, ensuring that the modified network contains no EGCs; for details see Materials and methods). It can be argued that some types of reactions should be preferentially removed; e.g., reactions only weakly supported by genomic evidence may be removed first, and it may be more likely that one direction of a reaction labeled as reversible represents a network error than that an irreversible reaction is erroneous. While the modified GlobalFit algorithm allows such differential weighing of different reaction types, we considered all reaction removals as equally likely in the application detailed below. Moreover, reactions could be preferentially removed depending on the estimated equilibrium constant (or standard Gibb’s free energy change ΔG_0_).

For 94% of metabolic models with EGCs (223 out of 238), GlobalFit found a set of reaction removals that eliminated all EGCs while maintaining the ability to produce biomass. In many cases, GlobalFit suggested the removal of the ATP synthase reaction. While this will indeed remove most ATP-producing cycles, it will also abolish the model’s natural ability to produce ATP through respiration. To avoid this undesired side effect, we performed a second search for reaction removals that eliminated all EGCs, this time forcing the algorithm to retain the ATP synthase reaction. This step could be adapted to the physiology of the studied organism by selecting a different reaction set to be retained. In each case, we could identify an alternative set of reaction removals; below, we only consider these alternative sets of suggested network changes. Note that GlobalFit does not actually remove the offending reactions, but constrains their fluxes to zero. This allows their reactivation in conditions where they are deemed thermodynamically feasible, although alternative measures must then be taken to avoid EGCs.

**Fig 4:**
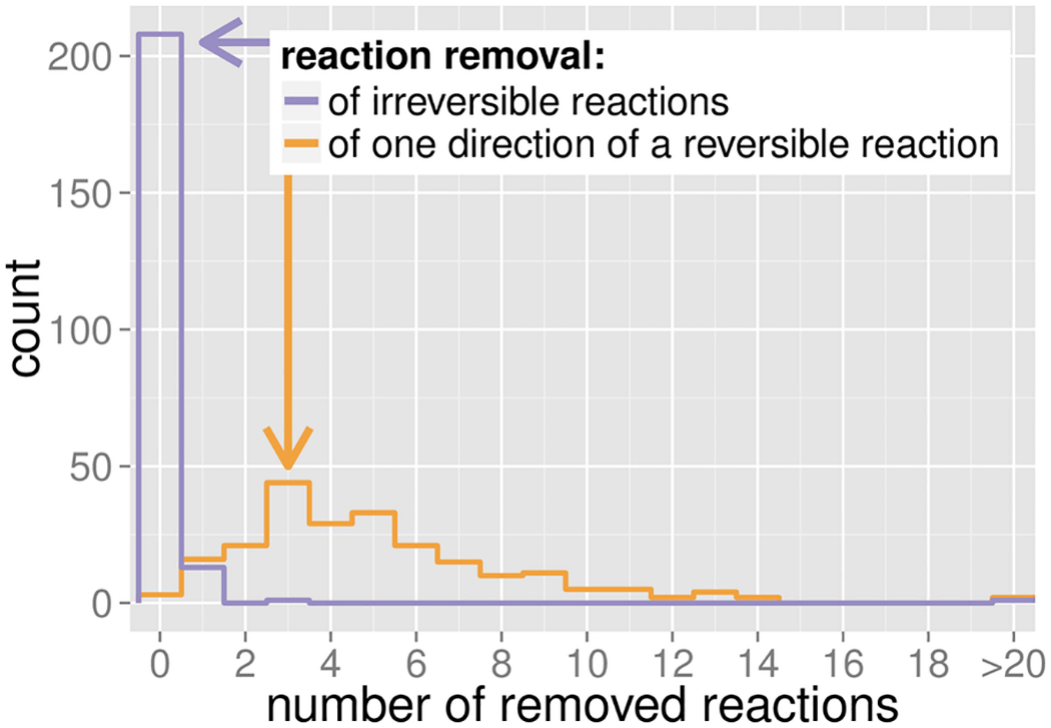
Most erroneous models can be corrected by making up to 5 originally reversible reactions irreversible. Purple: histogram of the number of irreversible reactions removed in each model to eliminate EGCs. Orange: histogram of the number of reversible reactions made irreversible to eliminate EGCs.

Most erroneous models can be corrected by making up to five originally reversible reactions irreversible (Fig 4). The removal of irreversible reactions was only rarely suggested by the algorithm (Fig 4), while the complete removal of reversible reactions was never observed. In the remaining unsolved models, EGCs could in principle be eliminated by adding reactions to the metabolic networks. The addition of reactions not directly connected to an EGC may be needed to restore biomass production in case no solution exists that preserves viability after EGC removal. While the modified version of GlobalFit is capable of suggesting such additions, the application of this strategy would require manual revision, as it might incorrectly add new metabolic capabilities.

While bi-level mixed integer optimization algorithms such as the one used by GlobalFit typically require long computation times, GlobalFit resolved most solvable EGCs in under 10s, and all but one EGC within one minute (Supplementary S1 Fig). The only calculation that required over one minute was for the yeastnet 7.6 model [27], for which the CPLEX solver did not find an optimal solution within the set limit of 60 hours on 16 CPUs. The best set of changes found for this yeast model eliminated all EGCs by removing 76 reactions (or reaction directions). According to CPLEX, there is no alternative elimination of EGCs with fewer than 33 reactions; thus, this model contains at least 33 EGCs. As many EGCs include transport reactions across cellular membranes, the large number of EGCs found in this eukaryotic model (and the resulting increased computation time) may be caused by the existence of several intracellular compartments and the associated transport processes.

**Fig 5:**
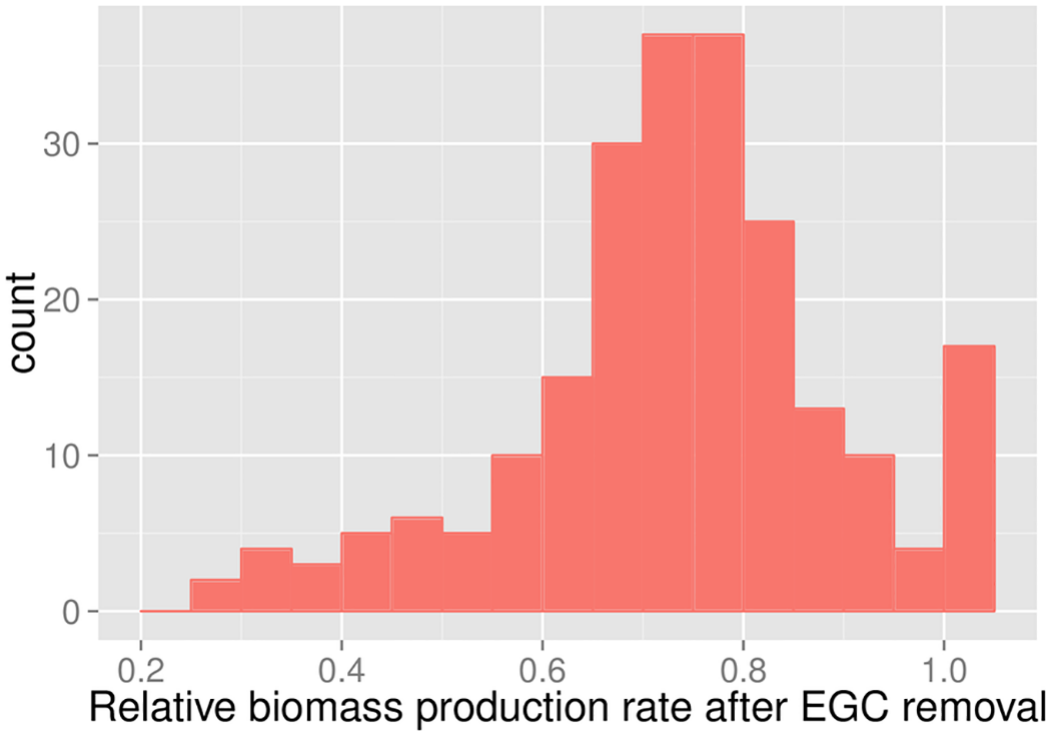
Removal of EGCs led to substantially reduced maximal biomass yield in most models. Histogram of the ratio between maximal biomass production rate before and after EGC removal.

Freely available energy may boost biomass production. Accordingly, the elimination of EGCs through the reaction removals suggested by GlobalFit resulted in biomass reductions in 92% of cases (206 out of 223), typically by more than 25% (Fig 5). This indicates that the *in-silico* biomass yield may be unrealistically high in a majority of automatically generated models.

### Examples for network corrections suggested by GlobalFit

**Fig 6:**
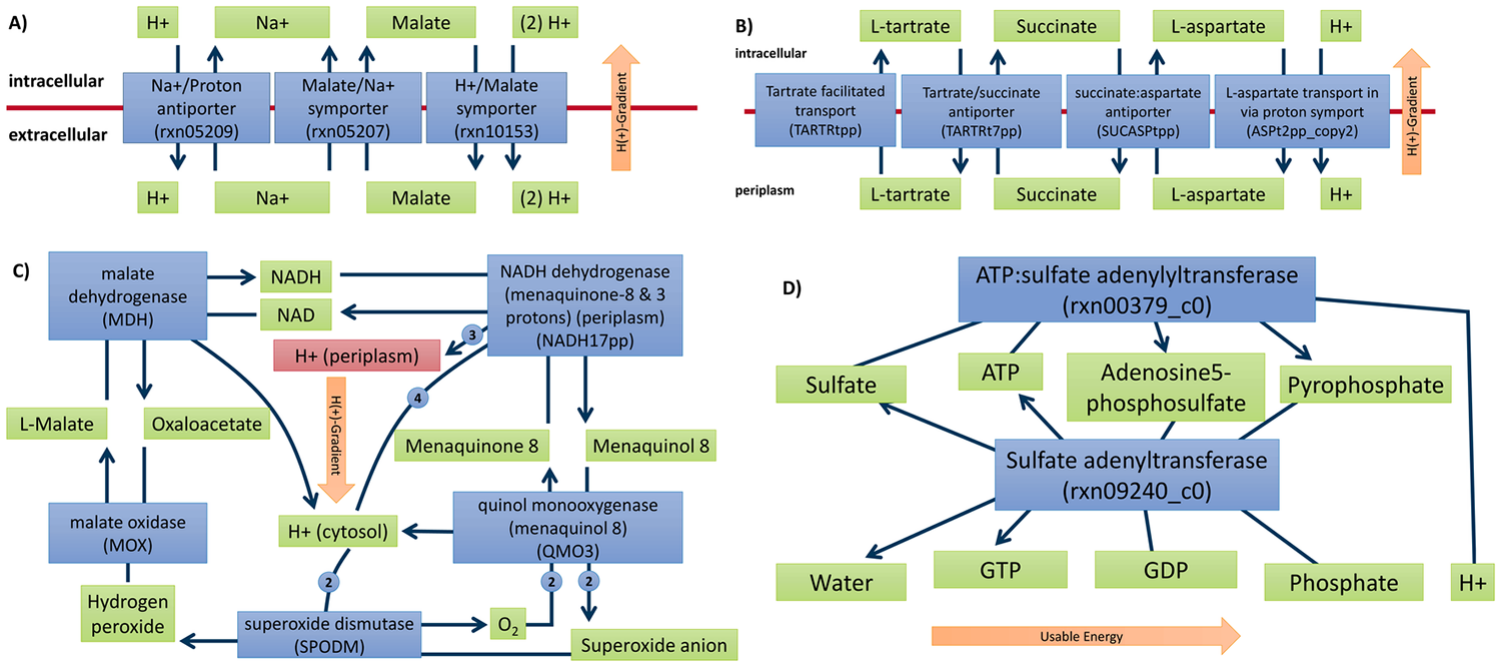
Examples of EGCs found in published genome-scale models. Green/red: metabolites; blue: reactions, linking substrates and products; orange: direction of the energy gradient utilized by the energy dissipation reaction. (A) The simplest identified cycle, which links a Na+/proton antiporter (exporting Na+ in exchange for a single proton) and a Malate/proton symporter (importing Malate together with two protons) via a Malate/Na+ symporter. (B) A cycle involving two antiporters and one symporter, driven by a transporter that translocates tartrate from the periplasm to the cytosol. (C) A NADH:menaquinone oxidoreductase, which translocates protons in the process of transferring electrons from NADH to Menaquinone 8, driven by a chain of four enzymes. (D) rxn00379 creates Adenosine 5'-phosphosulfate from ATP and sulfate. The equivalent sulfate adenyltransferase rxn09240 catalyzes the backward reaction, but charges a GTP in addition to the ATP.

One of the simplest EGCs we identified is displayed in Fig 6(A). This cycle is contained in only two metabolic models from The ModelSEED database, *Klebsiella pneumoniae* MGH 78578 (Seed272620.3) and *Flavobacterium johnsonia johnsoniae* UW101 (Seed376686.6). In this EGC, a malate symporter (rxn10153) transports malate together with two protons out of the cell. The exported malate molecule is then re-imported together with a sodium ion via the malate/Na+ symporter (rxn05207). The sodium is in turn exported by a Na+/Proton antiporter (rxn05209) in exchange for the import of only one of the protons of the first reaction. Thus, the second exported proton from the first reaction is free to drive an ATP-synthase reaction, generating ATP from ADP without access to an external energy source. To eliminate this EGC, the cost of either malate or sodium transport in terms of translocated protons must be corrected. This option was not given to GlobalFit, which instead suggests to remove the export direction of the malate symporter (rxn10153).

In the manually curated model iJO1366, six reactions (SPODM, SPODMpp, SUCASPtpp, SUCFUMtpp, SUCMALtpp, and SUCTARTtpp) were inactivated in the published model to avoid unrealistic energy generating loops by constraining their flux to zero [13]. We could identify two distinct EGCs (Fig 6B and 6C) by reactivating these reactions in the iJO1366 model and in the 54 other *E*. *coli* models derived from this reconstruction [25]. One of these EGCs is the rather simple cycle (Fig 6B) based on tartrate facilitated transport (TARTRtpp), found in 45 of the 55 *E*. *coli* strain reconstructions in Ref. [25]. This reaction spontaneously imports tartrate from the periplasm into the cell, while the tartrate/succinate antiporter (TARTRt7pp) exports tartrate, but simultaneously imports succinate. The cycle continues with the succinate/aspartate antiporter and then the aspartate/proton symporter, so that eventually a proton gradient between periplasm and cytosol is established. GlobalFit suggests to remove the utilized direction of the tartrate/succinate antiporter.

The other EGC found in the unconstrained *E*. *coli* models is a more complicated cycle (Fig 6C) that occurs in 46 of the 55 *E*. *coli* reconstructions [25], including the manually curated iJO1366 model [13]. A proton gradient across the periplasmic membrane is established by a NADH:menaquinone oxidoreductase (NADH17pp), which translocates protons in the process of transferring electrons from NADH to Menaquinone 8, driven by a chain of four enzymes, including superoxide dismutase (SPODM). In order to deactivate the cycle, GlobalFit removes the backward direction of the Malate oxidase (MOX) or the forward reaction of the Superoxide dismutase (SPODM). In this case, removal of the Malate oxidase would also be suggested by an analysis of standard free energy changes, at it is highly energetically unfavourable.

The EGC shown in Fig 6D was found in 99 out of 195 metabolic models from the ModelSEED database [20]. rxn00379 creates Adenosine 5'-phosphosulfate from ATP and sulfate. The sulfate adenyltransferase rxn09240 catalyses the backward reaction (and has the same EC-number assigned), but charges not only an ATP, but additionally a GTP in the process. To eliminate this EGC, GlobalFit suggests removing either one of the participating reactions.

### Conclusions

EGCs are a major issue in FBA modeling—they are able to produce energy out of thin air, thereby severely affecting the appropriate representation of energy metabolism and of biomass yield. EGCs not only affect the accurate representation of existing metabolic systems. They will be particularly problematic in evolutionary simulations that involve the incorporation of foreign metabolic reactions from other species [28–30]. Such mixing of reactions from disparate model reconstructions may easily introduce EGCs, and may thus lead to erroneous phenotype predictions. We have recently suggested a protocol for evolutionary simulations that avoids this problem [31]. Here, we present an improved computational method for the high-throughput identification of EGCs.

EGC identification is currently not a recognized step in model reconstruction, although some authors have eliminated EGCs from their manually curated models before publication. While constraint-based methods may avoid the utilization of EGCs based on thermodynamic considerations [17], such methods are computationally expensive and require careful analysis of the EGCs and the bounds on metabolite concentrations to guarantee the absence of EGCs from the resulting flux distributions. Instead, we propose to correct the metabolic model itself, and present a modified version of the previously published GlobalFit algorithm to eliminate EGCs through the removal of minimal reaction sets. The resulting model can then be used with the full suite of standard constraint- based methods.

We found EGCs in the majority of automatically generated models and in a small subset of manually curated networks. Many of the identified EGCs—in particular those that occurred most frequently—involved the erroneous maintenance of proton gradients across cellular membranes. The simplest EGCs would consist of two reversible reactions that catalyze the same biochemical conversion using different amounts of energy metabolites (Figs 2 and 6D). Such trivial EGCs are easily recognizable and are consequently rarely included in published metabolic networks; most EGCs in published models are more complex, and not easily identified by eye. We note that automatically reconstructed models often contain other types of errors as well [2]. For example, charge and mass imbalanced reactions appear to be common in automatic reconstructions and can lead to erroneous FBA predictions. Such reactions can potentially introduce EGCs, and we thus suggest to correct them as a preprocessing step.

The inclusion of reaction sets that are capable of forming an EGC into a metabolic network reconstruction is not necessarily erroneous. It is conceivable that one part of the cycle is thermodynamically feasible in one condition, whereas the other part is thermodynamically feasible in another condition, while both are not thermodynamically feasible simultaneously. Accordingly, modeling algorithms that respect thermodynamic constraints do not utilize potential EGCs [3, 6, 7, 17, 32–34]. FBA, however, does not consider thermodynamics; instead, optimization of its objective function (*e*.*g*., biomass production rate) will usually lead to the exploitation of EGCs. One possible solution would be to constrain the fluxes through thermodynamically impossible sections of EGCs to zero in each simulated environment; this, however, would require a detailed understanding of environment-specific thermodynamics (or, alternatively, environment-specific gene regulation).

Our algorithms are suitable to guide a manual curation of draft networks, and should be included in the standard toolbox used for metabolic network reconstruction. GlobalFit can enumerate alternative solutions to eliminate EGCs, which can then be used as a basis for expert curation. In the context of automated network reconstruction pipelines such as ModelSEED or kBase, our methods could be applied without human interaction, albeit at the risk of removing reactions that might be thermodynamically feasible in particular environments.

## Materials and methods

### Dataset and EGC detection

We started from 350 genome-scale metabolic networks (GSMs) that were downloaded from three databases: BiGG [22]–mostly manually created GSMs (accessed July 2015); ModelSeed [23]–GSMs created automatically from genome sequences (accessed July 2015); and MetaNetX [24]–a meta-database containing metabolic models from various sources (accessed January 2016). We removed networks that were unable to produce biomass in a maximally rich environment. We checked the correct direction of exchange reactions, and set the lower bound of the ATP maintenance reaction (ATPM) to zero, *i*.*e*., we did not require a non-growth-related production of ATP.

To each GSM, we added 15 energy dissipation reactions (Supplementary S1 Table), where the namespace for metabolite names had to match the source of the network, *i*.*e*., BiGG, ModelSEED, or MetaNetX. Because not every metabolic network covers the full range of metabolites used in the energy dissipation reactions (EDR), we checked the integration of the reactions in the network, defined as the fraction of the reaction’s metabolites also present in the remainder of the model (*i*.*e*., a reaction with an integration of 1 is completely integrated, whereas reactions with an integration < 1 cannot carry any flux). Because EGCs tend to run with maximal fluxes, all network reactions except the newly added ones (those in energy dissipation reactions) are restricted to fluxes in the range [–1, 1] for reversible and [0, 1] for irreversible reactions.

To establish the presence of EGCs for different energy metabolites, we maximized one energy dissipation reaction flux *v*_*d*_ at a time while prohibiting all influx into the model:

$$\text{max }(v_{d})$$

subject to:

$$\begin{array}{l} Sv=0\\ \forall i\notin E:\;v_{i}^{min}\leq v_{i}\leq v_{i}^{max}\\ \forall i\in E:\;v_{i}=0 \end{array}$$

Here, *S* is the stoichiometric matrix, ***v*** the vector of fluxes, *d* the index of one of the energy dissipation reactions, ***v***^*min*^ and ***v***^*max*^ the vector of lower and upper reaction bounds, respectively, and *E* is the set of indices of all exchange reactions.

An optimal value $v_{d}^{*}$ for this optimization with $v_{d}^{*}>\;0$ indicates the presence of at least one cycle that is able to generate a specific type of energy metabolite (corresponding to the index *d*) in the network. Because 0 ≤ ∣*v*_*i*_∣ ≤ 1 for all reactions other than dissipation reactions, the value of $v_{d}^{*}$ is a lower bound for the number of non-overlapping EGCs for the tested energy metabolite in the network.

### The modified GlobalFit algorithm

Once a GSM was identified to contain at least one EGC, GlobalFit was used to eliminate all EGCs from the network. GlobalFit was developed to find globally minimal sets of model changes that simultaneously reconcile sets of experimental growth and non-growth observations with model predictions; a detailed description of the original GlobalFit algorithm can be found in [26]. We modified GlobalFit for the efficient removal of EGCs as outlined below.

In this modified version, the only allowed type of model change is the removal of unidirectional reactions, where reversible reactions are treated as two independent unidirectional reactions. We contrast a single growth with a single non-growth case. The non-growth case reflects the removal of all EGCs: with no nutrient uptake allowed, the maximal sum of fluxes through the energy dissipation reactions must be zero, max(*∑*_*d*_∣*v*_*d*_∣) = 0. To ensure that reaction removals do not abolish biomass production by eliminating EGCs, we set up a growth case with a minimal biomass production rate in a rich medium that allows uptake of all nutrients.

Formally, we solve the following bi-level optimization problem, which is a variation of the original GlobalFit problem [26] (Variable definitions are listed in Table 1):

$${\text{min}}_{\delta }\left(\sum_{y\in M}\left(\delta_{y}^{RF}+\delta_{y}^{RB}\right)\right)$$ (1)

subject to:

$$S_{g}\;\times \;v^{g}=\;0$$ (2)

$$\forall_{y\in M}\;v_{y}^{min}\times \left(1-\delta_{y}^{RB}\right)\leq \;v_{y}^{g}\leq \;v_{y}^{max}\times \left(1-\delta_{y}^{RF}\right)$$ (3)

$$v_{\text{B}io}^{g}\geq \;T_{g}$$ (4)

$$S_{ng}\;\times \;v^{ng}\;=\;0$$ (5)

$$\forall_{y\in M}\;v_{y}^{min}\times \left(1-\delta_{y}^{RB}\right)\leq \;v_{y}^{ng}\leq \;v_{y}^{max}\times \left(1-\delta_{y}^{RF}\right)$$ (6)

$$\underset{v^{ng}}{\text{min}}\left(c^{t}\times \;v^{ng}\right)=0$$ (7)

**Table 1:** Definitions of the variables used in the system of equations that describes the modified GlobalFit algorithm.

*M*	The set of reactions included in the original (input) network reconstruction
*S*	Stoichiometric matrix of the original (input) network reconstruction
***v***	Flux vector
*g*	Growth case
*ng*	Non-growth case
$v_{y}^{min}$	Lower bound of reaction *y*
$v_{y}^{max}$	Upper bound of reaction *y*
$v_{Bio}^{g}$	Biomass reaction of the growth case
*T*_*g*_	Growth threshold of the growth case
***c***^*t*^	Vector containing ones and zeros. All entries are zero, except for the positions of the energy dissipation reactions

$\delta_{y}^{RB}$ and $\delta_{y}^{RF}$ are binary variables. Setting one of these variables to 1 will constrain the corresponding flux of the growth—Eq (3)—and non-growth case—Eq (6)–to zero. The total number of reaction removals is minimized, where the removal of forward and backward reaction is treated separately in Eq (1)–*i.e., $\delta_{y}^{RB}$* and $\delta_{y}^{RF}$ are independent. Both the growth and the non-growth case must be in steady state, Eqs (2) and (5). The biomass production of the growth case has to be greater than a predefined threshold T_g_, Eq (4). All entries in c^t^ are 0, except for the positions of the energy dissipation reactions, which are 1. The maximal summed flux through all energy dissipation reactions must be zero, Eq (7). We convert this bi-level optimization problem into a single level optimization problem as described in [26].

All calculations were run in GNU R with the SyBiL library [35] and a modified GlobalFit library [26] under linux. We used IBM ILOG CPLEX as the solver for the mixed integer linear optimizations. Each calculation was run on 8 CPU cores and 50GB main memory.

## Supporting information

S1 Fig Computation time. Distribution of computation (wall-clock) times for the application of GlobalFit to the metabolic models containing EGCs. While almost all computations finished in under a minute on a PC (8 CPUs, 50Gb RAM), the search for model corrections requires considerably more time for the yeast 7 metabolic network (data points off scale; the “simple” calculations were stopped after 61.27 hours and the “synthase” run needed 25.03 minutes). The red line (“simple”) is for runs allowing all reaction removals; the blue line (“synthase”) is for runs not allowing removal of the ATP synthase reaction.
(TIFF)

S1 Table Energy dissipation reactions. Energy dissipation reactions (EDRs) for each of the 15 different types of energy metabolites in the cell.
(XLSX)

S2 Table EGC occurrences in models. For each model, this table shows whether biomass production was possible at all (hasGrowth), whether energy generating cycles are present (hasEGCs), the identified types of EGCs (e.g., generates.ATP), and the reactions (or reaction directions) removed by GlobalFit for the “simple” run (all removals allowed) and with removal of the ATP synthase forbidden (“synthase”).
(XLSX)

S1 Text Toy model. A small example network that illustrates the inability of ll-COBRA and TMFA to reliably exclude EGCs.
(PDF)
