# Supplementary figures and images for: Erroneous energy-generating cycles in published genome scale metabolic networks: Identification and removal
Source: PLoS Comput Biol. 2017 Apr 18;13(4):e1005494. doi: 10.1371/journal.pcbi.1005494 (PMC5413070; doi:10.1371/journal.pcbi.1005494)

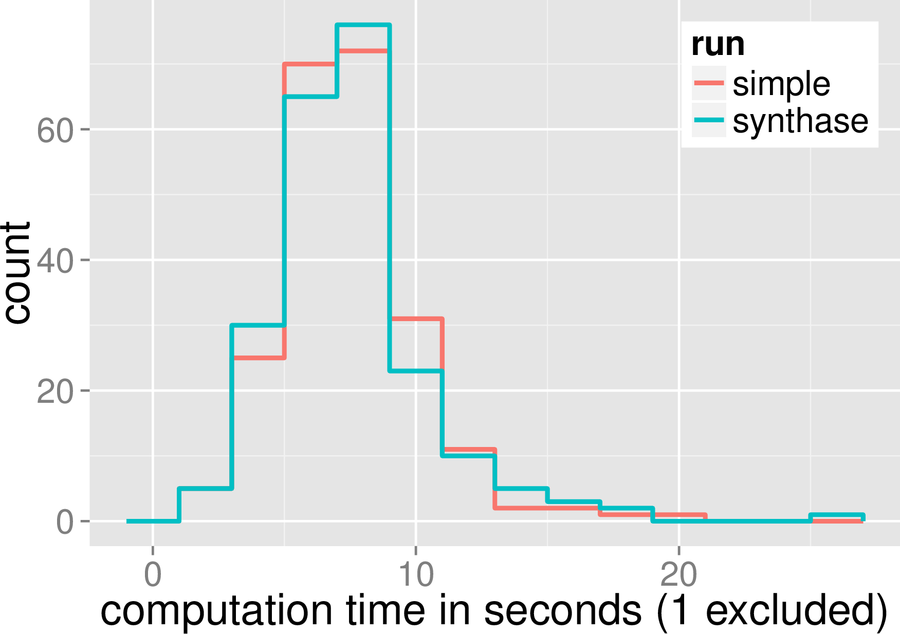

Supplement: S1 Fig — Distribution of computation (wall-clock) times for the application of GlobalFit to the metabolic models containing EGCs. While almost all computations finished in under a minute on a PC (8 CPUs, 50Gb RAM), the search for model corrections requires considerably more time for the yeast 7 metabolic network (data points off scale; the “simple” calculations were stopped after 61.27 hours and the “synthase” run needed 25.03 minutes). The red line (“simple”) is for runs allowing all reaction removals; the blue line (“synthase”) is for runs not allowing removal of the ATP synthase reaction. (TIFF) [file pcbi.1005494.s001.tiff]
