# Supplementary material for: Erroneous energy-generating cycles in published genome scale metabolic networks: Identification and removal
Source: PLoS Comput Biol. 2017 Apr 18;13(4):e1005494. doi: 10.1371/journal.pcbi.1005494 (PMC5413070; doi:10.1371/journal.pcbi.1005494)
Supplement: S1 Text — A small example network that illustrates the inability of ll-COBRA and TMFA to reliably exclude EGCs. (PDF) [file pcbi.1005494.s004.pdf]

Example Network

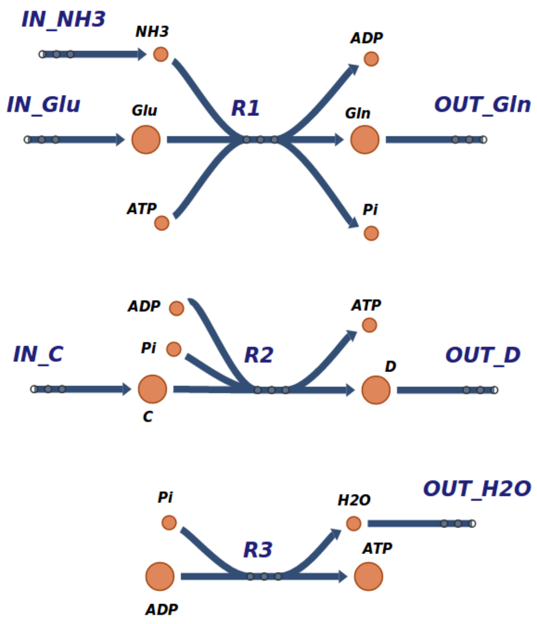

This example network consists of three reactions: R1: glutamine synthetase; R2: some arbitrary reaction converting metabolite C to metabolite D while phosphorylating ADP to ATP; R3: ATP synthase. *In vivo*, R1 and R2 follow the directions as depicted here. However, to mimic an error in the network, we deliberately assigned the wrong reaction direction to R3; the free energy change ΔG is positive under physiological conditions. The desired behaviour of the network should be to use metabolite C to make energy. This energy is then used to fuse ammonia and glutamate to glutamine. An FBA with this model that maximizes OUT_Gln could give one of three possible steady-state solutions: R1 and R2 active (an elementary flux mode), R1 and R3 active (the second elementary flux mode), or all three reactions active (a combination of the two previous elementary modes). When blocking the influx reaction IN_C, a correct model should return no outflux of glutamine. Instead, we get a solution with R1 and R3 active. ll-COBRA also returns this solution. This is because R1 and R3 do not form a type-III pathway, and thus ll-COBRA is unable to remove the cycle (Table S1_Text.1). Even TMFA is not able to reliably remove the cycle, as we can find an artificial distribution of metabolite concentrations (Table S1_Text.2) that leads to negative ΔG for both R1 and R3 (Table S1_Text.3); note, however, that this solution can be excluded in TMFA through appropriate bounds on the metabolite concentrations.

Table S1_Text.1: Given the sample network above and blocking the wrong reaction R3, FBA and ll-COBRA give correct reaction fluxes. However, introducing the wrong reaction R3 and blocking the influx of metabolite C (IN_C blocked) still results in a flux through the maximized reaction (OUT_Gln). Objective Reaction: blue; blocked reactions: red.

	**Correct Fluxes** (R3: blocked)		**Wrong Fluxes** (IN_C blocked)	
**Method**	FBA	ll-COBRA	FBA	ll-COBRA
**R1**	1000	1000	1000	1000
**R2**	1000	1000	0	0
**R3**	0	0	1000	1000
**IN_C**	1000	1000	0	0
**IN_Glu**	1000	1000	1000	1000
**IN_NH3**	1000	1000	1000	1000
**OUT_D**	1000	1000	0	0
**OUT_Gln**	1000	1000	1000	1000
**OUT_H2O**	0	0	1000	1000

Table S1_Text.2: Metabolite concentrations.

**Metabolite**	**ATP**	**ADP**	**Pi**	**H_2O**	**NH_3**	**Gln**	**Glu**
**Physiological Concentrations (mM)**	1.54	0.8	50	$55*10^3$	30	3.8	96
**Artificial Concentration (mM)**	10	10^5	10^5	$55*10^3$	10^3	0.01	10^3

Table S1_Text.3: Free energy changes for R1 and R3 calculated with equilibrator[1].

	**R1**	**R3**
**Physiological Concentrations**	-23.3 ± 1.6 [kJ/mol]	35.4 ± 0.6 [kJ/mol]
**Artificial Concentrations**	-9.3 ± 1.6 [kJ/mol]	-7.8 ± 0.6 [kJ/mol]

[1] http://equilibrator.weizmann.ac.il/
